# Deep Learning System for Recycled Clothing Classification Linked to Cloud and Edge Computing

**DOI:** 10.1155/2022/6854626

**Published:** 2022-11-03

**Authors:** Sun-Kuk Noh

**Affiliations:** SW Convergence Education Institute, Chosun University, Gwangju, Republic of Korea

## Abstract

Recently, IT technologies related to the Fourth Industrial Revolution (4IR), such as artificial intelligence (AI), Internet of things (IoT), cloud computing, and edge computing have been studied. Although there are many used clothing occurrences with 61 trillion worn of clothing consumption per year in Korea, it is not properly collected due to the efficiency of the used clothing collection system, and the collected used clothing is not properly recycled due to insufficient recycling system, lack of skilled labor force, and health problems of workers. To solve this problem, this study proposes a deep learning clothing classification system (DLCCS) using cloud and edge computing. The system proposed is to classify clothing image data input from camera terminals installed in various clothing classification sites in various regions into two classes, as well as nine classes, by deep learning using convolution neural network (CNN). And the classification results are stored in the cloud through edge computing. The edge computing enables the analysis of the data of the Internet of Things (IoT) device on the edge of the network before transmitting it to the cloud. The performance evaluation parameters that are considered for the proposed research study are transmission velocity and latency. Proposed system can efficiently improve the process and automation in the classification and processing of recycled clothing in various places. It is also expected that the waste of clothing resources and health problems of clothing classification workers will be improved.

## 1. Introduction

Recently, IT technologies related to the Fourth Industrial Revolution (4IR), such as artificial intelligence (AI), Internet of things (IoT), cloud computing, and edge computing have been studied. AI, led by machine learning and in-depth learning, has emerged as a key technology of 4IR. Various research and development related to AI are being carried out in various industrial fields. It is predicted that artificial intelligence will not be used only in the medical field, but will be used in personal (patient, general) health care along with the development of ICT technology.

Clothing image analysis technology has been applied to recommend or find specific clothing [1–5]. The analysis of clothing images includes fashion recognition that analyzes information about clothing images, and fashion retrieval that can find desired clothes from images. Among these clothing image analysis techniques, clothing recognition aims to analyze the categories and characteristics of clothing present in captured images, and there are FashionNet and Attentive Fashion Grammar Network based on the VGG-16 network applied to convolution neural network (CNN). In addition, although the utilization rate of recycled clothing is increasing in terms of environmental protection and resource saving, it is difficult to apply existing clothing recognition because of the deformation and overlapping phenomenon when the recycled clothing is carried out on the conveyor belt to be classified. However, a method of classifying recycled clothing using mass dataset and deep learning technology has been studied.

The textile industry consumes a lot of human resources in all processes such as raw material collection, dyeing, processing, and sewing, and the waste of resources and energy and the increase of environmental pollution are caused by the short-term waste of clothing produced through this process. For example, 1,500 liters of water is used to make a pair of jeans, and 10 to 15% of the chemicals that come out after weaving and removing dyes become wastewater and pollute the environment. If you buy clothes at a second hand shop, which sells used goods instead of buying new clothes, you can reduce environmental pollution as much. Therefore, there are many efforts in domestic and various countries to recycle and rewear waste clothing if possible. Although there are many used clothing occurrences with 61 trillion worn of clothing consumption per year in Korea [6], it is not properly collected due to the efficiency of the used clothing collection system, and the collected used clothing is not properly recycled due to insufficient recycling system, lack of skilled labor force, and health problems of workers.

Edge computing enables real-time decision making by minimizing delay time and bandwidth requirements because it can process more data in the network ‘edge' near the data source and reduce the data transmitted to the cloud data center. Cloud computing has its own advantages and importance for big data analysis and computation but have some disadvantages also in regards to real time and time sensitive data computation.

To improve the problem of data transmission networks, it is important that the combination of edge computing and cloud computing can provide you reduced disadvantages of cloud computing. IoT devices with edge computing and cloud computing will be able to run faster and process data efficiently without losing storage capacity and processing power. [7, 8].

In this study, to make an offer solution about the problem, I suggest a deep learning clothes classification system (DLCCS) using cloud and edge computing for efficient classification of recycled clothing. For the proposed study, first, the AI model AlexNet was used, IoT devices, camera and raspberry pie 3, was used, and clothing was classified into nine classes (Knit, Shirts, Jeans, etc.). The accuracy of the classification was measured and the usability of the proposed system was confirmed and announced [9]. The system proposed in this study is to classify clothing image data input from camera terminals installed in various clothing classification sites in various regions into two classes (top and bottom), or several classes by deep learning using CNN. And the classification results are stored in the cloud through edge computing. DLCCS can operate as edge computing, and edge computing can analyze the data of IoT devices on the edge of the network before transmitting to the cloud. The proposed system is expected to improve the process and automation efficiently in the classification and processing of recycled clothing. The remainder of this paper is organized as follows: in Section 2, the related work was reviewed. In Section 3, the suggestion of a system is presented. In Section 4, the simulation and results are presented. Finally, conclusions are given in Section 5.

## 2. Related Works

### 2.1. The Relationship between Clothing Industry and Environment and Resources

According to [10], 100 billion clothes are produced every year and 33 billion clothes are discarded. In the past 20 years, the number of people wearing clothes has not doubled, but the production of clothing has increased five times, and the annual purchase of clothes per person is about 68 clothes. In addition to creating clothing waste, the clothing business is also creating environmental and resource issues. Global warming is running out of water, and white cotton T-shirts have about 2,700 liters of water to make one sheet, which is the amount of water a person can drink for three years. In addition, the water consumed in the fashion industry with dye clothing accounts for one-fifth of the water consumed in the entire industry. The carbon emissions generated by making jean are 33 kg, which is 111 km long, and these jeans are made 4 billion in one year. And the amount of greenhouse gases that the clothing industry produces is more than the amount of greenhouse gases that the shipping industry and the aviation industry around the world produce. Of course, there are many zero-waist and eco-friendly brands and many good products are increasing recently, but the relationship between the clothing industry and the environment and resources is very big and important. In the reference, we can see how the clothes we put in the recycled clothing box are used in Korea and how the end of the clothes that were not selected until the end of the last one is used.

The clothes in the recycled clothing box are first collected by the old clothes collection box management company or individuals periodically (usually once every six days). These old clothes come in 40 tons per day to the collection company, and there are nearly 100 collection companies in Korea, that is, more than 4,000 tons of clothes are discarded in Korea per day. Of the clothes collected, only 5% of the clothes that are still commercial are distributed in ‘Vintage Shops' and ‘Relief Shops' in Korea and sold again, and all remaining 95% are exported overseas. Most of them are imported from developing countries, which is the fifth largest exporter of used clothing in the world, and they incinerate all clothes that are not really commercial.

### 2.2. Reuse Clothes

**Figure 1:**
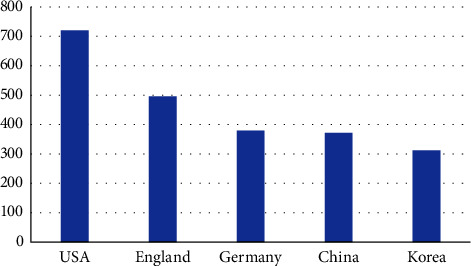
Exports of recycled clothes (2019 unit $ 1 million).

As shown in Figure 1, these are the countries that exported the most recycled clothing in 2019. [11]. In Korea, clothing waste is not included in the subject of separation collection, and separate discharge and collection are carried out in various forms by local governments.

Waste clothing collected by separation, and discharge is classified as reusable and impossible. Reusables are sold in flea markets, bazaars, etc., in Korea and exported to foreign countries including underdeveloped countries.

### 2.3. IoT, Edge Computing, and Cloud Computing

Edge computing is a distributed open architecture with distributed processing capabilities that enables mobile computing and IoT technology. The role of edge computing to date has been mainly used to collect, store, filter, and transmit data to cloud systems. Edge computing allows data from Internet of Things devices (terminals) to be analyzed at the edge of the network before being sent to the cloud [11–13]. Edge computing enables real-time decision making by minimizing delay time and bandwidth requirements because it can process more data in the network ‘edge' near the data source and reduce the data transmitted to the cloud data center, so that data is processed locally rather than cloud storage.

To simply define the most basic concept of cloud computers, one of the IT technologies of 4IR, when you do any work or work on a personal computer, simply input/output it to bring a program to a server called the cloud based on the Internet, and the analysis, processing, and management of the data are also format in the cloud space [14–16].

Cloud computing includes all ranges of IaaS (Infrastructure as a Service) that serves server resources to run servers, PaaS (Platform as a Service), which provides development platform middleware, and SaaS (Software as a Service) that serves applications.

**Figure 2:**
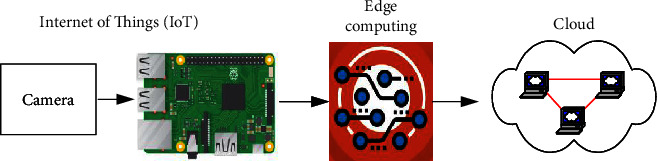
IoT and edge computing and cloud computing.

IoT and Edge computing and Cloud are shown in Figure 2. The IoT device consists of a camera and Raspberry Pi.

### 2.4. Deep Learning and CNN

AI, led by machine learning and in-depth learning, along with new technologies leading the 4IR, is applied through various research and development. Deep learning is a subclass of machine learning that uses artificial neural networks for tasks such as visual recognition and is used to train the computer to classify images based on clothing content. Deep learning neural networks include one or more hidden layers.

A CNN is used as a popular deep learning technique to analyze visual images to apply deep learning framework and is a neural network made by using the concept of a receptive field of a human visual neuron. CNN has a convolutional layer, pooling layer, and a rectified linear units as core elements. The convolutional neural layer is characterized by a weight kernel that sees the input and output in the form of a signal and shows the weight in the form of a small filter. The pulling of the nerve layer has the role of reducing the size of data, as it summarizes a few output values. And the effect of canceling the noise or the distortion of input data in this process may be obtained. The rectifying linear unit is a nonlinear neuron with the activity of a lamp function, and has the effect of simultaneously solving the calculation burden of the sigmoid function used in the neural network and the slope disappearance phenomenon in the reverse wave algorithm. In 2012, AlexNet is a CNN architecture and consists of eight learning, five convolutional, and three fully connected layers [17, 18]. Since 2015, the GoogLeNet and ResNet have been released and CNN's structure has continued to deepen and complicate [19, 20]. Among the latest CNN structures, the contents of the four classification algorithms are as shown in Table 1, such as AlexNet, VGG, GoogleLeNet, and ResNet.

### 2.5. Clothing Classification CNN

Research on CNN-based clothing recognition often focuses on fashion classification or detecting clothing by processing surveillance camera footage [21–26]. However, real-time clothing identification from surveillance videos is difficult in reliable clothing detection. Liu et al. introduced a robust clothing recognition and retrieval based on classification and a large-scale dataset. Lao et al. examined and presented the clothing attributes and type classification. In clothing classification with CNN using transfer learning AlexNet and GoogleNet, clothing image datasets are classified into several classes through deep learning [27–29].

**Figure 3:**
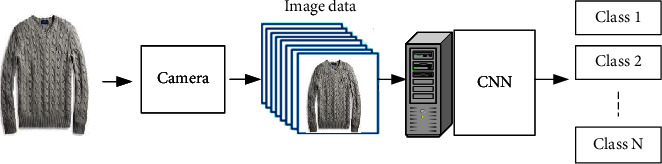
DLCCS.

**Figure 4:**
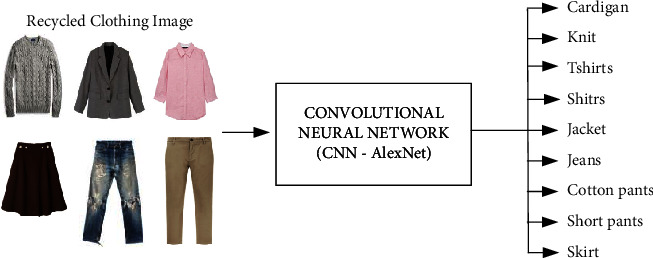
9-class clothing classification using CNN.

First, demonstrate clothing classification using large scale dataset, where the proposed model performs. Second, the results show that the model correctly classifies most of the test images with a success rate that is higher than 70%. Finally, we evaluate clothing classification using footage from surveillance cameras. The system performs well on this dataset, labelling about 65% of the test images correctly. The process of classifying the clothes image dataset using CNN is called a Deep Learning Clothes Classification System (DLCCS) and is shown in Figures 3 and 4.

## 3. Suggestion of System

**Figure 5:**
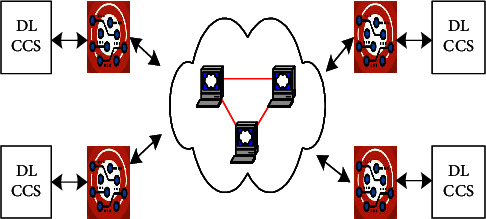
Concept of DLCCS using edge computing and cloud computing.

**Figure 6:**
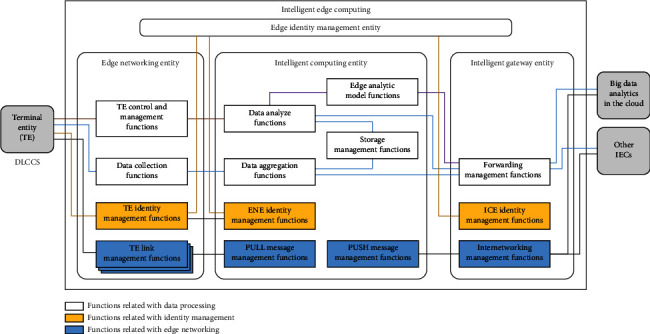
Architecture of DLCCS and edge computing and cloud computing.

**Figure 7:**
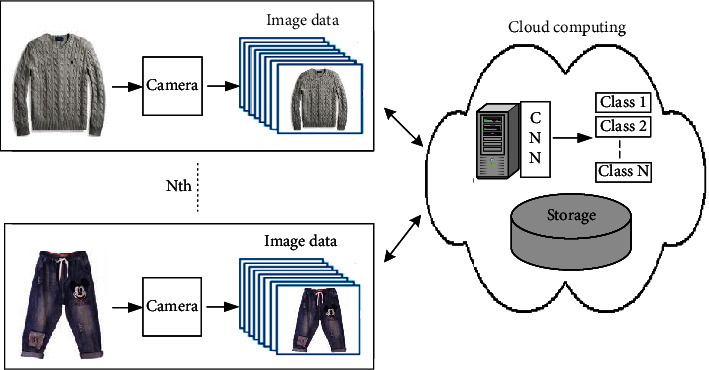
Suggestion of DLCCS using cloud computing.

The proposed system can be installed in various places where recycled clothing is collected, and the proposed system classifies the clothing image data set input from the terminal into several classes using CNN. Classes can be classified as large class and small classes. Large classes are top and bottom, adults and children, and small classes are knit, cardigan, coat, pants, skirt, etc. The classification results are transmitted and stored in the cloud through edge computing.  Figure 5 shows the structure in which distributed DLCCS is connected to edge computing and cloud networks. Clothing information classified in DLCCS is simulated considering optical communication in the case of wired and 5G in the case of wireless as each data transmission path. ITU-T Q.5001 signalling requirements and architecture of intelligent edge computing [30], the architecture is shown in Figure 6. The suggestion of DLCCS using cloud computing is shown in Figure 7. CNN implemented in cloud computing processes image data sent from IoT devices. However, cloud computing has problems such as cost increase, hacking, and virus.

**Figure 8:**
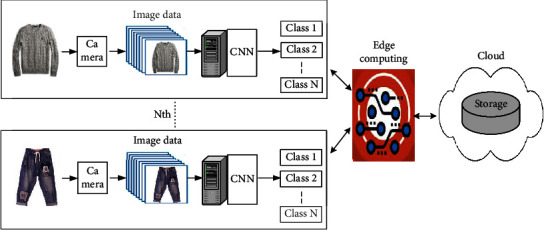
Suggestion of DLCCS using edge computing and cloud computing.

To solve this problem, many DLCCS are composed of IoT devices and CNN and are connected to cloud using Edge computing to build a recycled clothing classification network. Edge computing is a technology that processes data in real time near the site where the data is generated, and it is the opposite concept of cloud computing that processes data through servers on the Internet. Suggestion of DLCCS using Edge computing and Cloud computing is shown in Figure 8. CNN, implemented as edge computing in DLCC, processes, classifies, and stores image data transmitted from IoT devices installed at the place where recycled clothing is classified and stores it in cloud storage.

In Figure 7, delay time increases as the physical distance between IoT devices and cloud computing increases. In Figure 8, edge computing is applied to enable data processing, so that the delay time and bandwidth according to data transmission and storage can be drastically reduced. In addition, personal information and security problems can be solved because anonymous and encryption of the collected data is possible. This will make it closer to real-time to receive data analysis (clothing classification) results, while reducing the burden on crowd data centers (storage). In addition, the proposed system can process the image data of the clothing classification work site and send notifications about abnormalities, malfunctions, etc., to the manager when an intelligent sensor is installed in the IoT device.

## 4. Simulation and Results

**Figure 9:**
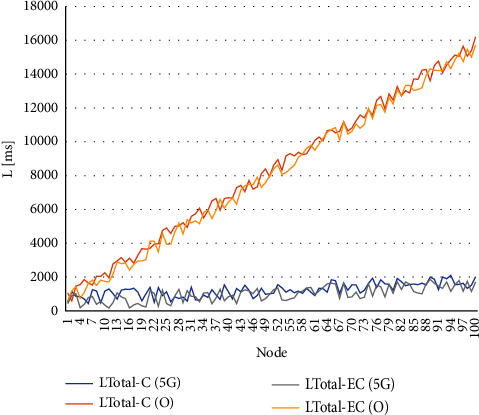
Simulation results of *L*_Total-C_ and *L*_Total-EC_.

**Figure 10:**
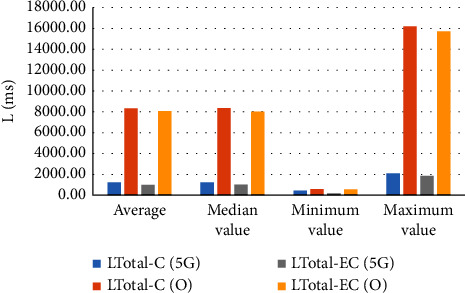
Simulation results analysis.

The simulation is shown in Figures 9 and 10, assuming two transmission networks and measuring the data transmission delay time. Figure 7 shows the configuration in which clothing image data is sent to cloud computing from distributed IoT devices, classified and stored by AI. Figure 8 assumes that clothing is classified in IoT devices distributed by AI and data is stored in the cloud through edge computing. As shown in Figure 9 and Table 1, the total latency (*L*_Total-C_) is *L*1+*L*2+*L*3. Delay time (*L*_1_) is generated when the clothing image information is transmitted from the node to the cloud, and the deep learning classification and judgment time (*L*_2_) are added to the cloud, and the delay time (*L*_3_) is generated to return the result to the node again. In Figure 9, after the deep learning, classification and judgment are made in the node, only the results are sent to the cloud and the storage results are received again, so the total latency (*L*_Total-EC_) is reduced. In particular, the delay in the gateway existing in the network channel and the congestion delay between each node will be added as the number of nodes increases. In the case of a wired channel, an optical network was simulated, and in the case of a wireless channel, a 5G network was simulated. The channel is wired using MATLAB, the simulation conditions are shown in Table 2, and the results are same as Figure 9, and the latency of the proposed method is reduced. In Table 2, assuming that the random delay time of random variables follows the Gaussian probability distribution, the DLPT time was applied by the arithmetic mean of the CNN model measurement results of the reference document [9, 31].

Computing the network channel model for simulation is as follows:

(1) $$\begin{array}{c} L_{Total-C}\left(n\right)={L_{1}+L}_{2}+L_{3}=\overset{n}{\underset{i=1}{\sum }}kN_{i}+\overset{n}{\underset{i=1}{\sum }}{RD}_{i}+DLPT,k=k_{o},k_{5G}\text{,} \end{array}$$

(2) $$\begin{array}{c} L_{Total-EC}\left(n\right)=L_{1}+L_{2}=\overset{n}{\underset{i=1}{\sum }}{kN}_{i}+\overset{n}{\underset{i=1}{\sum }}{RD}_{i},k=k_{o},{\;k}_{5G}\text{.} \end{array}$$

In Figure 9, the simulation results of L_Tolal-C_ by using equation (1) and the simulation results of L_Tolal-EC_ using equation (2) were shown. With the same number of nodes, the total latency of the optical wired network environment (ko) increased compared to the 5G wireless network environment (k_5G_), but the increase in congestion due to the increase of nodes showed that the wireless network environment was more frequent. The result of equation (1) was obtained by adding DLPT to the result of equation (2), which indicates that L_Tolal-C_ has more latency than L_Total-EC._

In Figure 10, as a result of the simulation, the latency of the channel has the greatest effect on the total latency. And when comparing the optical network and the 5G network, it was confirmed that the total latency (*L*_Total-EC_) was smaller with the configuration of the proposed model using the 5G network. On average, *L*_Total-EC_ (5G) was reduced about 8 times compared to *L*_Total-EC_ (O).

## 5. Conclusions

Recently, IT technologies related to the Fourth Industrial Revolution (IR) such as artificial intelligence (AI), Internet of Things (IoT), cloud computing, and edge computing have been studied. As AI technology is gradually developing, the demand for machine learning work with smart devices is increasing rather than remote data centers, and edge computing will be an essential technology for AI-based Internet of Things. Edge computing is a technology that complements the shortcomings of cloud computing. Edge computing and cloud computing coexist and various services are being provided to complement each other.

In Korea, recycled clothing is not properly collected due to the inefficiency of the used clothing collection system, and the recovered clothing is not properly recycled due to the lack of recycling system. In this paper, I proposed a deep learning clothing classification system using cloud and edge computing to classify recycled clothing. Instead of classifying and storing clothes using CNN in the cloud as shown in Figure 7, DLCCS classifies clothes using AI at the part (edge) where image data (class) is generated as shown in Figure 8 and stores them in the cloud. In other words, the proposed DLCCS classifies clothes (classes) using CNN on the clothes image data set input from the IoT, and stores the classification results in the cloud through edge computing. In addition, multiple DLCCS can establish a single recycled clothing sorting cloud network.

In the DLCCS configuration of Figures 7 and 8, the total delay time generated in information transmission was simulated, and it was confirmed that the proposed model of Figure 8 had lower overall delay time than the model of Figure 7. And the average latency time was about eight times lower for LTotal-EC (5G) than for LTotal-EC (O).

Because sorting recycled clothing requires a small amount of data and fast processing, edge computing is used, and a lot of data obtained in this process is accumulated in the cloud with high computing power. Therefore, the proposed DLCCS system can be integrated into the cloud for sorting and processing recycled garments in multiple locations to efficiently improve the process. In addition, in garment sorting workshops, the proposal system can send notifications of abnormalities or malfunctions to managers when intelligent sensors are installed in IoT devices. It is also expected to improve the wastage of clothing resources and the health problems of clothing sorting workers.

## Figures and Tables

**Table 1:** Classification algorithms-AlexNet, VGG, GoogleLeNet, and ResNet.

Classification algorithms	Contents
AlexNet	(i) First large scale CNN
	(ii) Designed in parallel to perform parallel operation with two GPUs
	(iii) Consists of 5 conv layers and 3 FC layers
	(iv) Use ReLU as an activation function

VGGNet	(i) Eight layers on AlexNet, but 16–19 on VGGNet, making the network much longer and using smaller filters
	(ii) Used a tiny filter of 3 × 3 size only
	(iii) The lowest efficiency because of the large memory usage and the large amount of operation

GoogLeNet	(i) Implementation of local topology with the concept of “network within a network”
	(ii) 22 layers that can be learned with weights
	(iii) Stacking and making several inception modules
	(iv) Classification of ImageNet classes using softmax

ResNet	(i) Using a method called residual learning
	(ii) A very deep network depth of 152 layers
	(iii) Degradation problem with lower performance from 20 layers or more
	(iv) Each residual block consists of two 3 × 3 conv layers
	(v) Memory usage and computation are moderate, but the accuracy is high

**Table 2:** Simulation condition.

Simulation				Figure 5	Figure 6
Node (*N*)				100	100
*L* (ms)	*L*1	Channel latency (*k*) (ms)	Optical network (*k*_o_)	150	150
			5G network (*k*_5G_)	10	10
	*L*2	Random delay time (RD) (ms)	Gaussian distribution	Random variables	Random variables
	*L*3	Deep learning model process time (DLPT) (ms)	Parallel processing	227	—

## Data Availability

Previous reported data were used to support a part in this study and these prior studies are cited at relevant places within the text as references [9, 25–28].
